# Use of Artificial Intelligence in the Advancement of Breast Surgery and Implications for Breast Reconstruction: A Narrative Review

**DOI:** 10.3390/jcm12155143

**Published:** 2023-08-06

**Authors:** Ishith Seth, Gabriella Bulloch, Konrad Joseph, David J. Hunter-Smith, Warren Matthew Rozen

**Affiliations:** 1 Department of Plastic Surgery, Peninsula Health, Melbourne, VIC 3199, Australia; 2 Faculty of Medicine, The University of Melbourne, Melbourne, VIC 3053, Australia; 3 Faculty of Medicine, The University of Wollongong, Wollongon, NSW 2500, Australia

**Keywords:** artificial intelligence, AI, breast reconstruction, breast surgery, advancement

## Abstract

Background: Breast reconstruction is a pivotal part of the recuperation process following a mastectomy and aims to restore both the physical aesthetic and emotional well-being of breast cancer survivors. In recent years, artificial intelligence (AI) has emerged as a revolutionary technology across numerous medical disciplines. This narrative review of the current literature and evidence analysis explores the role of AI in the domain of breast reconstruction, outlining its potential to refine surgical procedures, enhance outcomes, and streamline decision making. Methods: A systematic search on Medline (via PubMed), Cochrane Library, Web of Science, Google Scholar, Clinical Trials, and Embase databases from January 1901 to June 2023 was conducted. Results: By meticulously evaluating a selection of recent studies and engaging with inherent challenges and prospective trajectories, this review spotlights the promising role AI plays in advancing the techniques of breast reconstruction. However, issues concerning data quality, privacy, and ethical considerations pose hurdles to the seamless integration of AI in the medical field. Conclusion: The future research agenda comprises dataset standardization, AI algorithm refinement, and the implementation of prospective clinical trials and fosters cross-disciplinary partnerships. The fusion of AI with other emergent technologies like augmented reality and 3D printing could further propel progress in breast surgery.

## 1. Introduction

Artificial Intelligence (AI) is an umbrella term encompassing various facets of computer learning [1]. AI models have the potential to improve accuracy and efficiency in clinical practice and improve patient-centered outcomes [2]. The first clinical use of AI in surgery occurred in 1985 during an AI-assisted robotic neurosurgical biopsy procedure [3]. We have since witnessed an extraordinary evolution of these technologies within the healthcare sector, which extends to a myriad of diagnostic and decision-making procedures [4]. Machine learning (ML) is a subset of AI that utilizes algorithmic tools to discern patterns within datasets and make future predictions without requiring explicit programming [5,6]. ML integration into surgery began in the early 21st century [7] and has profoundly impacted the surgical field, supporting developments in the planning, navigation, and analysis of surgical procedures [8]. Modern improvements in computational capabilities and the emergence of big data analytics have allowed AI to expand its efficiency and applicability to medicine and healthcare at an unprecedented pace [9,10].

Furthermore, in the AI realm, two main categories discussed are narrow and broad AI [11]. Narrow AI is designed to perform a specific task under a limited set of constraints, while broad AI learns and applies knowledge across a wide range of tasks at a level equivalent to or beyond that of humans. In the context of healthcare, this states that general AI would be capable of performing any intellectual tasks that a human doctor can carry out. However, this type of AI remains largely theoretical. Deep learning (DL) is a subset of ML that mimics human learning by utilizing artificial neural networks (ANN) [12]. ANN comprises artificial neurons/nodes that process information similar to the human brain. Each node has interconnected links with specific weights and thresholds; when a node’s output surpasses a defined threshold, it is activated, transmitting data to the network’s subsequent layer. If it falls short, no data progression occurs. ANN represents the leading edge of AI technology in surgery and has a potential role in many aspects of healthcare, from identifying complex patterns in datasets to making predictions about disease progression.

Breast reconstruction is an essential element in the multidisciplinary management of breast cancer and aims to restore patient body image and post-mastectomy quality of life [13,14]. AI technologies show promise with respect to impacting surgical disciplines like breast reconstruction pre-, intra-, and post-operatively by assisting otherwise subjective interpretations and decision making, which risk inconsistencies in surgical outcomes and long-term complications [15,16].

**Figure 1:**
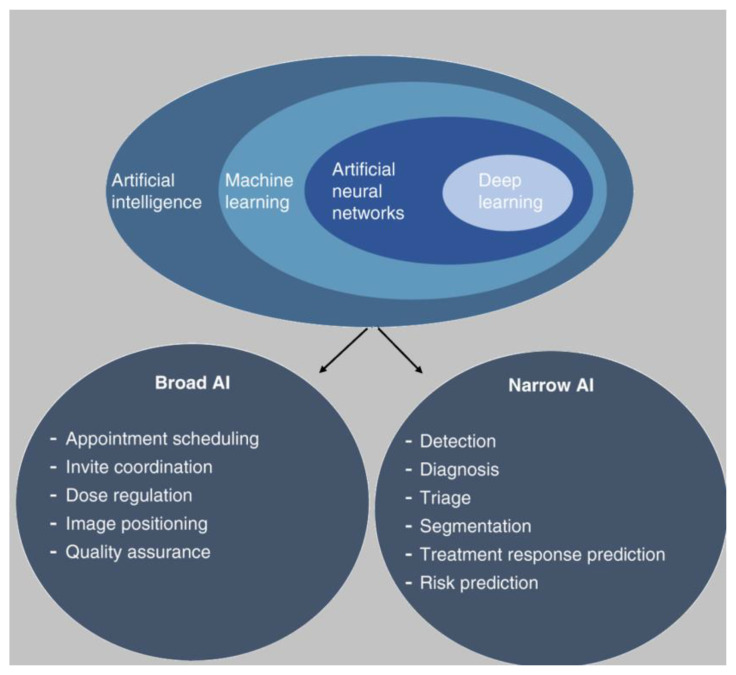
Applications of artificial intelligence in breast reconstruction surgery.

The potential applications of AI within breast surgery are extensive, and surgeons should stay abreast of developments to improve their clinical practice and optimize patient outcomes. In breast reconstruction, narrow AI systems can be utilized in analysing mammograms, ultrasounds, and MRI images to identify potential anomalies, and AI algorithms can optimize personalized surgical techniques and help predict patient-specific outcomes (Figure 1) [17]. Meanwhile, broad AI can assist in the integration of data from genetics, radiology, pathology, and clinical history to create a comprehensive patient profile in order to formulate personalized treatment plans, predict potential complications, and ensure quality assurance [18,19]. However, a review of how AI specifically impacts breast reconstruction has yet to be carried out. This review further explores the application of AI in breast surgery, inspects the benefits of its use in breast reconstruction via the critical assessment of existing research, and speaks to the future of this fast-evolving subject.

## 2. Materials and Methods

Two independent authors (IS and GB) searched for relevant studies on Medline (via PubMed), Cochrane Library, Web of Science, Google Scholar, Clinical Trials, and Embase databases from January 1901 to June 2023. The search terms consisted of “artificial intelligence”, “AI”, “machine learning”, “deep learning”, “breast”, “reconstruction”, “breast surgery”, and “breast cancer surgery”. The search strategy for PubMed was “Artificial Intelligence” [Title/Abstract] OR “AI” [Title/Abstract] OR “Machine Learning” [Title/Abstract] OR “Deep Learning” [Title/Abstract] AND “Breast” [Title/Abstract] OR “Breast Reconstruction” [Title/Abstract] OR “Breast Surgery” [Title/Abstract] OR “Breast Cancer Surgery” [Title/Abstract]. The search strategy was designed to encapsulate all studies that investigated AI in breast surgery. In addition, the reference lists of previous reviews and articles were manually checked for further articles that are relevant to the subject. All studies included were in or translated into English.

**Figure 2:**
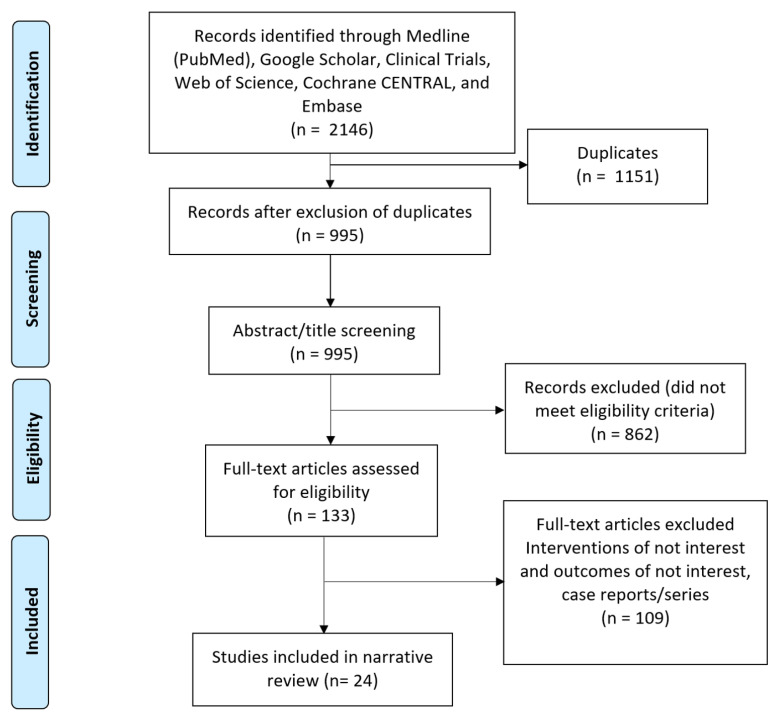
PRISMA flow diagram of selected studies.

The inclusion criteria were English language and human studies that investigated AI and breast surgery reconstruction from 1901 to June 2023. The acceptable study designs were review articles, randomized controlled trials, prospective studies, and retrospective studies. The exclusion criteria were articles such as commentaries, editorials, case studies, and studies not written in English or not investigating the use or implications of AI in breast reconstruction. The PRISMA study selection flow diagram is shown in Figure 2.

## 3. Results

### 3.1. AI and Detection of Breast Tumors from Imaging

The primary functions of AI in breast cancer screening currently involve object detection and tumor classification as benign or malignant according to the Breast Imaging Reporting and Data System (BIRADS), which was traditionally performed by human experts. These approaches underscore the comprehensive role AI plays in improving disease detection and treatment outcomes (Figure 1). In a multi-center study conducted by Hamyoon et al., ML was utilized to examine 1288 breast lesions based on BIRADS and morphometric features [20]. The model achieved an area under the receiver operating characteristic curve (AUC) of 0.885, outperforming both expert radiologists and radiology residents who achieved AUCs of 0.814 and 0.632, respectively, across all cohorts.

Radiomics is an approach to medical imaging that relies on AI to analyse quantitative information extracted from images [21]. It operates on the premise that these extracted features reflect activities that occur at the genetic and molecular levels. Radiomics is classified as supervised or unsupervised. Supervised ML starts with training AI using pre-existing data archives, whereas unsupervised ML categorizes information without reference to pre-existing data or data derived from the image itself. DL models recognize and classify images by autonomously reducing each to a set of numeric features that are processed using a multilayer neural network. Kallenberg et al. were among the early adopters of DL for breast cancer risk assessment. They used a convolutional sparse autoencoder coupled with a simple classifier, successfully linking the identified features to breast cancer in a significant case–control classification performance [22]. This was achieved by training and testing the model on contralateral mammographic images from two distinct databases. Concurrently, Li et al. effectively differentiated between high-risk groups and healthy controls by applying a pre-trained AlexNet model to mammographic images [23]. In another innovative approach, Gastounioti et al. harnessed convolutional neural networks to merge parenchymal complexity measurements into distinct meta-features for risk prediction [24]. Their method outperformed conventional parenchymal pattern analysis, further underscoring the potential of such groundbreaking techniques to enhance breast cancer risk prediction. These studies present early evidence that full-field digital-mammography-based DL models may prove more accurate than traditional density-based and epidemiology-based models in predicting breast cancer risk.

In 2017, Becker et al. performed a retrospective study using neural network image analysis software for mammography diagnostics. Their model was tested on two datasets (n = 178) and demonstrated comparable performance with radiologists but with better sensitivity [25]. In 2020, a study by McKinney et al. evaluated an AI breast cancer screening system on an international scale [26]. They found that using it for breast cancer screening improved prediction performance by reducing both type 1 and type 2 error rates whilst simultaneously reducing physician workload. In 2021, Raya-Povedano et al. performed a retrospective evaluation of AI-based breast cancer screening strategies. In an examination of 15,987 mammograms, they found that AI system implementation would simultaneously increase cancer detection sensitivity and reduce human workload [27]. These findings support the theoretical benefits of implementing AI in the detection of breast lesions and, therefore, have significant implications for breast reconstruction procedures.

### 3.2. Preoperative Use of AI

Once a breast lesion has been detected, AI algorithms can assist surgeons in preoperative planning by assessing patient-specific factors, such as breast volume, shape, and symmetry. Preoperative imaging studies are essential for assessing the vascular supply of the breast and can determine the reliability of reconstructive techniques [28]. Specific ML technologies, like the Faster-RCNN with Inception-ResNet-v2 deep-learning framework for ultrasound breast images, have further optimized these processes and show potential for use in surgical interventions.

Research highlighting the benefits of AI support in the preoperative period of breast reconstruction has been performed. In 2020, Mavioso et al. evaluated the feasibility of using computer software to support preoperative planning for microsurgical reconstruction using the DIEP flap technique. The researchers developed a convolutional neural network to identify perforators and tested the software on 40 patients. Results showed that the software was able to detect key perforators in 97.7% of cases, further supporting the idea that ML techniques can improve the preoperative planning of breast reconstruction [15].

AI can also be used to simulate results during the preoperative period of breast surgery, providing clinicians and patients alike valuable insights into likely cosmetic outcomes. In 2022, Chartier et al. evaluated a neural network that, after training itself on real clinical images, was able to consistently generate preoperative images that were comparable to real surgical results [29].

### 3.3. Intraoperative Use of AI

Within the confines of the operating theater, AI has the potential to support surgeons via real-time decision making and enhance surgical precision, but for the present, these have not been explored with respect to breast reconstruction surgery. In endoscopic surgeries, computer vision algorithms could scrutinize intraoperative imagery, highlighting crucial anatomical structures and offering guidance for meticulous tissue dissection and implant placement. Additionally, in orthopedic surgery, AI-powered robotic systems augmented surgical dexterity, reducing the risk of complications and improving surgical outcomes [30]. Although autonomous systems have achieved clinical application in orthopedic and neurosurgical fields [31], the role of AI-assisted robotics for surgical intervention on deformable breast tissue remains theoretical at present.

### 3.4. Postoperative Use of AI

AI-based systems can help optimize postoperative care via the early detection of complications. By carrying out clinical monitoring and the evaluation of medical imagery, AI algorithms can autonomously detect the symptoms of infection, hematoma, or mispositioned implants. This would in turn facilitate timely intervention and decrease the likelihood of enduring complications. In 2021, Myung et al. validated the ability of machine learning models and identified factors that predict breast reconstruction complications in 568 cases. They found that AI technologies could be effectively used for assessing the risk of negative patient outcomes in reconstructive breast surgeries [32].

AI has also been used to facilitate comprehensive patient assessment and help forecast postoperative pain [33]. In 2020, Nair et al. developed an ML system that was able to predict postoperative opioid requirements in patients undergoing ambulatory surgery, further optimizing postoperative care [33]. These systems offer significant improvements over conventional methods, such as clinician-collected questionnaires, and provide advantages within a healthcare system where time and human resources are often constrained. By improving the efficiency and quality of postoperative pain assessments and prediction models, AI has the potential to help prevent debilitating post-mastectomy pain syndromes in patients who undergo breast surgery. In 2020, Juwara et al. tested the ability of AI to predict neuropathic pain after breast cancer surgery. After testing on a cohort of 204 patients, the authors concluded that ML models can identify predictors for neuropathic pain in patients who have undergone breast surgery better than traditional methods [34].

Kenig et al. recently developed an ad hoc neural network to identify key breast features for breast symmetry evaluation. The study used 200 frontal photographs of patients who underwent breast surgery and tested the neural network on 47 frontal images of patients who underwent breast reconstruction after breast cancer. The neural network was successful at localizing key breast features, including breast boundaries and the nipple–areolar complex in 97.7% of cases, with an impressive mean detection time of 0.52 s [16]. By automating the detection of key breast features, AI could revolutionize the evaluation of breast symmetry post-reconstruction [16].

## 4. Discussion

AI can play a crucial role in advancing breast reconstruction by facilitating preoperative planning, surgical precision, personalizing reconstructions, and aiding postoperative care. AI predictive models, utilizing preoperative data, assist surgeons in decision making and patient outcome predictions. AI also enhances surgical simulations by integrating with radiographic imaging to create accurate 3D models, streamlining preoperative planning and intraoperative navigation, and contributing to robot-assisted surgery. In terms of patient-specific reconstructions, AI leverages ML algorithms to customize implants, enhancing postoperative results and patient satisfaction. AI’s role in selecting the reconstruction type and predicting the aesthetic results of different techniques further assists decision making. AI’s postoperative contributions include predicting potential complications and enabling more accurate surgical outcome evaluations.

A major application of AI in breast surgery aims towards predicting patient outcomes, aligning with the broader utilization of AI in contemporary surgical practice. ML is frequently coupled with traditional statistical modeling for predictions, where it consistently demonstrates superior performance [34]. This was particularly evident in a longitudinal study carried out by Huang et al., which showcased the ability of AI to forecast 5-year mortality rates following breast cancer surgery [35]. Their research validated the predictive abilities of multiple ML models on a dataset of 363 patients, finding the artificial neural network to be the most accurate. A 2019 retrospective analysis by Kalafi et al. surveyed the clinical data of 4902 patients with pathologically confirmed breast cancer and found that ML methods produce desirable survival prediction accuracy [36]. These results highlight the utility of AI in the prediction of patient outcomes and the impact that AI can have on the selection of the best treatment protocols.

Sadly, the current role of AI in intraoperative breast reconstruction surgery is an untouched area of research. However, advancements in other surgical areas point to its potential benefits if they were extrapolated. For example, the conventional pathology sectioning of breast tumors can impede operating room efficiency, but Haifler et al. showed that the ML analysis of spectroscope images on a benchtop setting could differentiate renal cell carcinoma from benign renal tissues with high accuracy, sensitivity, and specificity (92.5%, 95.8%, and 88.8%, respectively) [37] while simply optimizing camera positioning could lead to better surgeon visualization and reduced operator error [38]. The combination of ML with augmented reality (AR) technologies may also enhance the safety and quality of surgeries [39], with these having been demonstrated in prostate cancer and limb reconstruction surgeries [40,41]. For example. Porpiglia et al. evaluated mpMRI data during AR robot-assisted radical prostatectomies in 30 patients, which facilitated effective procedures [41]. Similar approaches may be of benefit in breast surgeries, in which precise soft tissue resection is also integral to outcomes. The integration of AI with AR technologies such as the HoloLens™ (Redmond, WA, USA) has also implicated benefits in extremity reconstruction and could assist in the intraoperative identification of suitable perforators for breast reconstructive surgeries [42].

While this review presents evidence supporting the theoretical applications of AI and its branches, it is important to understand that these may not always be clinically applicable [42]. Many of the models discussed in these publications depend on curated datasets that are far more navigable than those typically available in clinical practice, and the absence of diverse and representative datasets creates difficulties in training robust and universally applicable AI models. The algorithms underlying their outputs tend to be complex and cryptic to the unfamiliar eye, and the current complexity of AI necessitates specialized knowledge and expertise for effective implementation, making them difficult to comprehend. The learning mechanisms of some systems are also often hard to replicate. Strategic programming, the utilization of "Explainable ML", and comparisons with clinical gold standards may serve as potential solutions to this challenge. Other numerous challenges have to be overcome, such as data quality, privacy considerations, and ethical issues, to ensure the integration of AI into clinical practice. The integration of AI technologies into clinical workflows and surgeon acceptance also play critical roles that need thorough consideration.

Beyond technical complexities, AI raises ethical concerns, including potential job losses, economic implications, and the lack of human touch. Legal, ethical, and social issues, such as the absence of regulatory structures for AI technology, must also be resolved. Economic factors and the availability of such technology in low- and middle-income countries warrant consideration. AI is still in its experimental stage, and it is imperfect and prone to various flaws. The ethical issues associated with the use of AI in breast surgery need to be further discussed and resolved [43].

To fully harness the use of AI in breast surgery, collaboration among clinicians, researchers, and industry experts is necessary. Future research should prioritize the creation of standardized datasets, the optimization of AI algorithms, and the execution of prospective clinical trials to validate the efficacy and safety of AI-based approaches. Interdisciplinary collaborations can facilitate the integration of AI into clinical practice and promote widespread adoption. Current research offers a foundational understanding of AI and highlights both realized and potential benefits for its usage. The clinical application of AI models, however, remains relatively limited and novel. Future approaches must prioritize clinical relevance to encourage data-driven patient–clinician decision-making in breast surgery. Prospective randomized studies to assess the impact of AI in clinical practice are warranted for these models to reach their full potential.

The combination of AI with other emerging technologies shows promise for developments in breast surgery. The study by Chae et al. using MRI angiography data to superimpose scans on patients using AR headsets found it useful for assessing breast morphology, which is in turn relevant for reconstruction [44]. AR applications have been shown to enrich the planning of perforator flaps in breast reconstructive surgeries [34]. Three-dimensional printing in breast reconstruction provides another potential area for AI contribution, as 3D printing can aid patient decision making and surgical planning in breast reconstruction [45], and the AI-optimization of 3D printing has been demonstrated for select medical applications [46]. Additionally, the autonomy of robotic AI models is constrained by parameters such as task complexity, environmental difficulties, and the level of human independence [30], and the intraoperative application of AI for robotic surgeries is currently in its infancy [29]. Further investigations, including validation studies and clinical trials, should be pursued to ensure the safety and efficacy of incorporating these tools into healthcare practice.

## 5. Conclusions

The evidence base supporting the use of AI within the field of breast surgery is growing. Its uses transcend simple breast lesion screening, and the benefits of its implementation have been demonstrated across pre- and postoperative periods. Current research elucidates the specific benefits of AI in breast reconstruction and supports further research in this area. This research identifies several promising avenues for future investigations in this dynamic field, promising advantages for both healthcare providers and patients.

## Data Availability

Data are available from the corresponding author upon reasonable request.
